# The ability of SABRE, a new quantitative receptor function model, to quantify receptor binding from even challenging concentration-effect data with a single unified fit

**DOI:** 10.3389/fphar.2026.1715771

**Published:** 2026-01-29

**Authors:** Barbara Olah, Vera Tarjanyi, Gabor Viczjan, Ignac Ovari, Andras Csoto, Zoltan Szilvassy, Bela Juhasz, Judit Zsuga, Rudolf Gesztelyi, Tamas Erdei

**Affiliations:** 1 Department of Orthodontics, Faculty of Dentistry, University of Debrecen, Debrecen, Hungary; 2 Doctoral School of Nutrition and Food Sciences, University of Debrecen, Debrecen, Hungary; 3 Department of Pharmacology and Pharmacotherapy, Faculty of Medicine, University of Debrecen, Debrecen, Hungary; 4 Institute of Plant Protection, Faculty of Agricultural and Food Science and Environmental Management, University of Debrecen, Debrecen, Hungary; 5 Department of Psychiatry, Faculty of Medicine, University of Debrecen, Debrecen, Hungary

**Keywords:** atrium, curve fitting, functional data, inotropy, K_d_, receptor affinity, SABRE model

## Abstract

The Signal Amplification, Binding affinity, and Receptor-activation Efficacy (SABRE) model is the most recent general and quantitative model of receptor function. A specific extension of the SABRE model enables the determination of K_d_ (the equilibrium dissociation constant of the agonist-receptor complex) and q (the fraction of receptors remaining operable after pretreatment with an irreversible receptor antagonist) from exclusively functional data. In the present investigation, we reevaluated the concentration-effect (E/c) data of our related recent study on the SABRE model to assess the properties of our newly developed multiline model, inspired by professional criticism of our previous study in question. We have found this multiline model, constructed within the framework of the SABRE model, to be capable of providing reliable results *via* one global fitting (i.e., with a single unified fit), even for our somewhat challenging data (containing some uncertainty). The multiline model that proved to be the most suitable for the current data was a relatively complex, six-model global fitting. These results further emphasize the significance of finding the best way to fit the equations of the SABRE model to the functional data to be evaluated.

## 1 Introduction

Modelling the function of receptors (in a pharmacological sense) can be useful in solving practical problems and may also provide valuable information about the underlying mechanisms of biological phenomena (Motulsky and Christopoulos, 2004; Kenakin, 2022). The most recent general and quantitative receptor function model is the Signal Amplification, Binding affinity, and Receptor-activation Efficacy (SABRE) model (Buchwald, 2017; Buchwald, 2019; Buchwald, 2020; Buchwald, 2022; Buchwald, 2023; Buchwald, 2025a). In addition to providing a tool to understand and simulate experimental phenomena, this model can be used to obtain estimates for K_d_ (the equilibrium dissociation constant of the agonist-receptor complex) and q (the fraction of the operable receptors after partial irreversible receptor inactivation) from purely functional data, specifically from appropriately constructed concentration-effect (E/c) curves (Buchwald, 2022).

In a previous study from our laboratory, we aimed to demonstrate the utility and main features of the SABRE model by evaluating E/c data generated with three A_1_ adenosine receptor full agonists in isolated, paced guinea pig left atria, before and after a pretreatment with an irreversible A_1_ adenosine receptor antagonist (Olah et al., 2025). In that study, we developed four fitting strategies of the SABRE model and compared them with one another. Finally, we recommended a two-step procedure (consisting of the third and fourth fitting strategies) as the best way to evaluate the presented sort of data (Olah et al., 2025).

Soon after, the results of our above-mentioned study (Olah et al., 2025) were commented on by Buchwald (2025b) and a single-step procedure was suggested as a more appropriate manner to achieve the same goal. We have found the core idea of this improvement noteworthy. Thus, we hereby propose our own new multiline model, designed for the current version of the GraphPad Prism software, that uses the same nomenclature and “fitting logic” as before (Olah et al., 2025), but incorporates the improvement suggested by Buchwald (2025b). Accordingly, we have named this multiline model the “fifth” fitting strategy, as a continuation of the four fitting strategies described in our previous work (Olah et al., 2025).

## 2 Methods

### 2.1 The reevaluated data

In the present study, we reevaluated the E/c data that were used in a recent study (Olah et al., 2025) and were originally generated in an earlier investigation from our laboratory (Gesztelyi et al., 2013). These E/c curves were constructed with NECA (5′-(*N*-ethylcarboxamido)adenosine), CPA (*N* ^*6*^-cyclopentyladenosine) and CHA (*N* ^*6*^-cyclohexyladenosine), generally considered to be A_1_ adenosine receptor full agonists (Fredholm et al., 2001; Deb et al., 2019). The E/c curves were constructed in the absence (labelled “N”) and presence (labelled “X”) of a pretreatment with FSCPX (8-cyclopentyl-*N* ^*3*^-[3-(4-(fluorosulfonyl)benzoyloxy)propyl]-*N* ^*1*^-propylxanthine), an irreversible A_1_ adenosine receptor antagonist (Srinivas et al., 1996). Thus, we worked with “Furchgott-type” data, i.e., E/c data obtained at different levels of the operable receptors (Furchgott, 1966; Furchgott and Bursztyn, 1967).

For curve plotting and fitting, the independent variable (X value) was the common logarithm of the molar concentration of the agonists. To obtain the dependent variable (Y value), the percentage decrease in the initial contractile force of the isolated and paced guinea pig left atria was first determined (E), and then this effect value was expressed as the percentage of the maximal effect (E_max_) achieved in the given experimental system during the given study (E/E_max_ %). Thus, in fact, we created E/E_max_ % vs. logc curves but, for simplicity, we called them E/c curves in this investigation as well.

### 2.2 The fifth fitting strategy, i.e., our newly proposed multiline model (constructed within the SABRE model)

Based on the suggestion of Buchwald (2025b), our new recommendation for fitting our “Furchgott-type” E/c data is the following six-model global fitting, presented as a multiline model designed for GraphPad Prism (RRID:SCR_002798):

NECA=100*ε_NECA*γ*10^n*X/ε_NECA*γ−ε_NECA+1*10^n*X+10^n*logKd_NECA)

NECAq=100*q*ε_NECA*γ*10^n*X/q*ε_NECA*γ−q*ε_NECA+1*10^n*X+10^n*logKd_NECA)

CPA=100*ε_CPA*γ*10^n*X/ε_CPA*γ−ε_CPA+1*10^n*X+10^n*logKd_CPA

CPAq=100*q*ε_CPA*γ*10^n*X/q*ε_CPA*γ−q*ε_CPA+1*10^n*X+10^n*logKd_CPA)

CHA=100*ε_CHA*γ*10^n*X/ε_CHA*γ−ε_CHA+1*10^n*X+10^n*logKd_CHA)

CHAq=100*q*ε_CHA*γ*10^n*X/q*ε_CHA*γ−q*ε_CHA+1*10^n*X+10^n*logKd_CHA

<A>Y=NECA

<D>Y=NECAq

<B>Y=CPA

<E>Y=CPAq

<C>Y=CHA

<F>Y=CHAq

**FIGURE 1:**
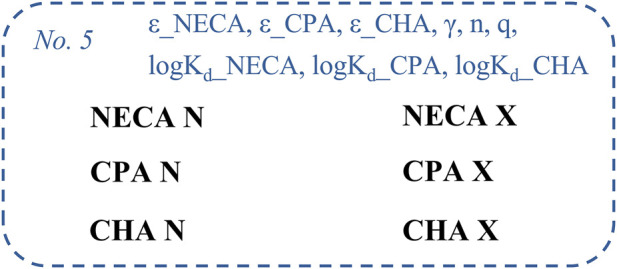
The fifth fitting strategy of the SABRE receptor function model applied to six data sets, consisting of E/c curves of three synthetic A_1_ adenosine receptor full (or close to full) agonists (NECA, CPA, CHA), constructed in isolated, paced guinea pig left atria, in the absence (“N”) or presence (“X”) of a pretreatment with FSCPX, an irreversible A_1_ adenosine receptor antagonist. The SABRE parameters, which were shared among the data sets (at least before their constraint to unity), are marked in blue. E/c, concentration-effect; SABRE, Signal Amplification, Binding affinity, and Receptor-activation Efficacy; NECA, 5′-(*N*-ethylcarboxamido)adenosine; CPA, *N* ^*6*^-cyclopentyladenosine; CHA, *N* ^*6*^-cyclohexyladenosine; FSCPX: 8-cyclopentyl-*N* ^*3*^-[3-(4-(fluorosulfonyl)benzoyloxy)propyl]-*N* ^*1*^-propylxanthine.

Here, the six-model global regression means that a separate equation should be fitted to each data set (six equations for the E/c curves of the six experimental groups of the original investigation: Gesztelyi et al., 2013). For this curve fitting, some parameters were shared among some data sets (Figure 1; Table 1). In the final regression, n and the three ε parameters were constrained to unity (to improve the reliability of the other estimates), as previously (Olah et al., 2025). Importantly, a single fitting can provide all estimates for the parameters.

**TABLE 1:** Results provided by the SABRE model using the fifth fitting strategy (i.e., by fitting the multiline model presented).

Parameters	NECA N	NECA X	CPA N	CPA X	CHA N	CHA X
ε parameters	= 1.00					
n	= 1.00					
γ	85.43					
	44.29 to 254.20					
	0.9833					
logK_d_ parameters	−5.84 *−5.88*		−6.09 *−5.93*		−5.38 *−5.51*	
	−6.12 to −5.37 *−6.40 to ?*		−6.38 to −5.62 *−6.42 to ?*		−5.66 to −4.91 *−5.84 to −4.88*	
	0.9372 *0.9870*		0.9495 *0.9840*		0.9441 *0.9717*	
K_d_	1.4 µM *1.3 µM*		0.8 µM *1.2 µM*		4.2 µM *3.1 µM*	
q	0.22 *0.22*					
	0.17 to 0.27 *0.18 to 0.27*					
	0.6058 *0.5047*					
R^2^	0.9878	0.9329	0.9833	0.9127	0.9684	0.9734
Gl. R^2^	0.9603					
Adj. R^2^	0.9597					

**FIGURE 2:**
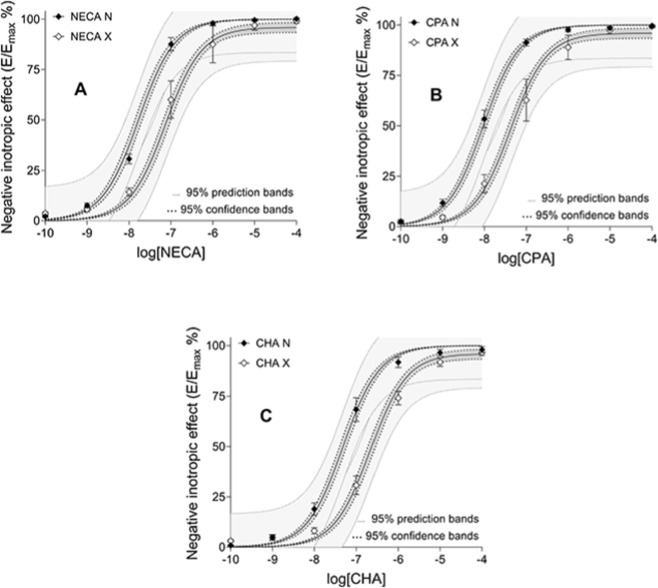
The E/c curves of three A_1_ adenosine receptor full agonists, NECA (panel **(A)**), CPA (panel **(B)**) and CHA (panel **(C)**), generated in isolated, paced guinea pig left atria in the absence (filled symbols) and presence (open symbols) of a pretreatment with FSCPX, an irreversible A_1_ adenosine receptor antagonist. The x-axis shows the common logarithm of the molar concentration of the given agonist, while the y-axis denotes the direct negative inotropic effect expressed as a percentage of the maximal effect achieved in this system (±SEM). The continuous lines show the best-fit curves of the SABRE model, fitted according to the fifth fitting strategy. For more details (sharing, constraints), see: Table 1. The thick dotted lines indicate the 95% confidence bands, while the thin dotted lines represent the 95% prediction bands. E/c, concentration-effect; SABRE, Signal Amplification, Binding affinity, and Receptor-activation Efficacy; NECA, 5′-(*N*-ethylcarboxamido)adenosine; CPA, *N* ^*6*^-cyclopentyladenosine; CHA, *N* ^*6*^-cyclohexyladenosine; FSCPX, 8-cyclopentyl-*N* ^*3*^-[3-(4-(fluorosulfonyl)benzoyloxy)propyl]-*N* ^*1*^-propylxanthine.

The reevaluated E/c curves were constructed with three A_1_ adenosine receptor full agonists (NECA, CPA, CHA) in the absence (“N”) and presence (“X”) of partial irreversible inactivation of the A_1_ adenosine receptors (for more details, see: Figure 2). During the final regression, the parameters related to ε (ε_NECA, ε_CPA, ε_CHA) and n were fixed at unity, while γ, q and the parameters related to logK_d_ (logK_d__NECA, logK_d__CPA, logK_d__CHA) were shared among all data sets (where the given parameters occurred). For each variable parameter, the best-fit value (top), confidence interval (middle) and dependency value (bottom) are presented. For comparison, logK_d_ and K_d_ values obtained with the third fitting strategy, and q provided by the fourth fitting strategy are also shown *in italics* from our previous study (Olah et al., 2025). E/c, concentration-effect; NECA, 5′-(*N*-ethylcarboxamido)adenosine; CPA, *N* ^*6*^-cyclopentyladenosine; CHA, *N* ^*6*^-cyclohexyladenosine; SABRE, Signal Amplification, Binding affinity, and Receptor-activation Efficacy; ε, the receptor-activation efficacy characterizing the agonist-receptor complex; n, a Hill-type coefficient characterizing the postreceptorial signaling; γ, the gain factor of the postreceptorial signaling; K_d_, the equilibrium dissociation constant of the agonist-receptor complex that characterizes the binding affinity; q, the fraction of receptors, which ones remained operable after the pretreatment with the irreversible antagonist; R^2^, the (“individual”) coefficient of determination; Gl. R^2^, the global coefficient of determination; Adj. R^2^, the adjusted global coefficient of determination.

The parameters characterizing the postreceptorial signal handling (γ and n) apply to all data sets, while the parameters describing the particular agonist-receptor interactions (ε_NECA, ε_CPA, ε_CHA, logK_d__NECA, logK_d__CPA and logK_d__CHA) refer only to the data set pairs generated with the same agonist. Furthermore, the parameter characterizing the efficiency of the pretreatment with FSCPX (q) applies only to the three data sets that underwent this pretreatment. For the classic appearance of the multiline model, see: Supplementary Appendix.

### 2.3 Data processing and presentation

Curve plotting and fitting were implemented with GraphPad Prism 10.6.1 for Windows (GraphPad Software Inc., La Jolla, CA, USA).

The precision of regression was characterized by the width of the 95% confidence interval (CI) of the best-fit values. For computing 95% CIs, the “asymmetrical” option was always chosen. The precision of the curve fitting and the precision of the E/c curve data were characterized by the distance of the best-fit curve from the corresponding 95% confidence bands and by the 95% prediction bands, respectively.

When setting the way in which the software checks how well the experimental data define the model, the option “Identify ambiguous fits” was chosen.

The degree to which each variable parameter was intertwined with all the others was indicated by dependency, the value of which could range from 0 (independent parameter) to 1 (redundant parameter). Dependency values greater than 0.9 and 0.99 could be considered high and unacceptably high, respectively.

The goodness of fit of the model was quantified by the “individual” and global values of the coefficient of determination (R^2^) and the adjusted value of the global R^2^. This adjusted global R^2^ is much lower than the global R^2^ if the model contains redundant parameters (Graphpad, 2025).

## 3 Results

The logK_d_ and q values provided by the fifth fitting strategy were similar to the corresponding values obtained previously using the third fitting strategy (Olah et al., 2025). Moreover, the related reliability measures (95% confidence limits and dependency) showed some improvement owing to the fifth fitting strategy (Table 1). The γ and (especially) q values (plus the related reliability measures) determined with the fifth fitting strategy were also close to the corresponding data obtained with the fourth fitting strategy (Olah et al., 2025) (Table 1). The measures characterizing the fitting as a whole (the different sorts of R^2^) and the appearance of the best-fit curves and their confidence and prediction bands were also similar to those presented in our original work (Olah et al., 2025) (Table 1; Figure 2).

## 4 Discussion

In the present study, we have improved our previous work (Olah et al., 2025) by elaborating a fifth fitting strategy incorporating Buchwald’ suggestion, the core idea of which is that each agonist-related parameter should be individualized (for the specific agonist used: NECA, CPA and CHA) (Buchwald, 2025b). Thus, instead of ε and logK_d_, ε_*agonist* and logK_d__*agonist* (e.g., ε_NECA and logK_d__NECA) should be fitted. The curve fitting software, used here, allows the E/c curves generated with a particular agonist to be fitted only to the equation that contains the corresponding agonist-related parameters. This maneuver is similar to the one that allows the E/c curves without and with an FSCPX pretreatment to be fitted separately (and adequately). In this way, six equations can be obtained (as a combination of the three agonists and the two outcomes of the FSCPX pretreatment). The global nature of regression can be ensured by sharing the agonist-related parameters (ε_*agonist* and logK_d__*agonist*), γ, n and q.

In fact, the six equations in question were almost ready as the fourth fitting strategy (Olah et al., 2025). To finalize these equations, we replaced the nonspecific parameter ε and the specific logK_d_ values of the three agonists with the appropriate individualized parameters (ε_NECA, ε_CPA, ε_CHA, logK_d__NECA, logK_d__CPA and logK_d__CHA). To ensure a single unified fit, all these individualized agonist-related parameters were shared among the data sets (in addition to γ, n and q). The fitting constraints, which assigned each of the six equations to the corresponding one of the six data sets, could be left the same for the fifth strategy as for the fourth strategy.

## 5 Conclusion

Our present work has demonstrated that the most recent method for estimating K_d_ values of agonist-receptor complexes from E/c data (Buchwald, 2022), an application of the SABRE receptor function model (Buchwald, 2017; 2019), is capable of providing reliable results through one global fitting (i.e., with a single unified fit), even for our data with some challenge. This challenge stemmed from the fact that our E/c curves, constructed in the presence of an irreversible antagonist, did not show a decrease in their maximal effect (as compared to the corresponding control E/c curves), rendering these “Furchgott-type” data somewhat uncertain. Our results further emphasize the importance of finding the best way to fit the equations of the SABRE model to this type of functional data. In the present case, this “best way” was a properly constructed multiline model (i.e., a six-model global fitting).

## Data Availability

The datasets presented in this study can be found in online repositories. The names of the repository/repositories and accession number(s) can be found in the article/Supplementary Material.
