# Supplementary material for: The ability of SABRE, a new quantitative receptor function model, to quantify receptor binding from even challenging concentration-effect data with a single unified fit
Source: Front Pharmacol. 2026 Jan 29;17:1715771. doi: 10.3389/fphar.2026.1715771 (PMC12894327; doi:10.3389/fphar.2026.1715771)
Supplement: Supplementary file 2 [file DataSheet1.docx]

# **Appendix**


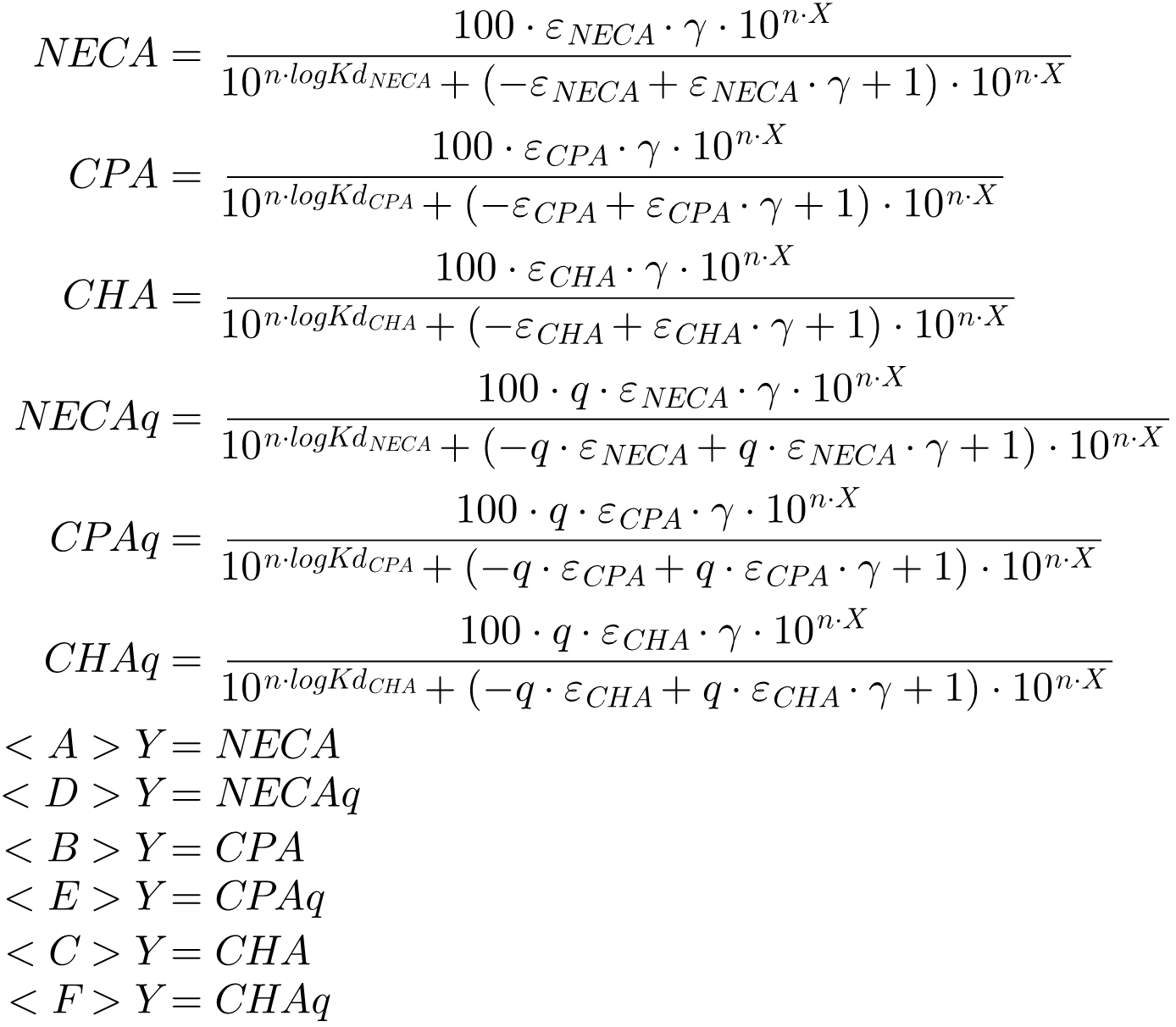


The multiline model, consisting of 12 lines (rows), of the fifth fitting strategy in an image automatically generated by the fitting software. The first six rows represent the SABRE model (Buchwald, 2022) tailored for E/c data obtained with three agonists (NECA, CPA, CHA) at two different levels of the operable receptors. In these equations, “NECA”, “CPA” and “CHA”, furthermore “NECAq”, “CPAq” and “CHAq” refer to the dependent variable (Y value) of the equation to be fitted to the E/c data constructed with the named agonist, without, furthermore with a pretreatment with an irreversible antagonist, respectively. The second six rows (from <A> to <F>) are constraints that assign each of the first six equations to the corresponding one of the six E/c data sets (distinguished with column headers from A to F in the database used). SABRE: Signal Amplification, Binding affinity, and Receptor-activation Efficacy; E/c: concentration-effect; NECA: 5′-(*N*-ethylcarboxamido)adenosine; CPA: *N^6^*-cyclopentyladenosine; CHA: *N^6^*-cyclohexyladenosine; q: the fraction of receptors remaining operable after pretreatment with an irreversible receptor antagonist
